# Impaired adenosine-induced myocardial perfusion in children with mild/moderate aortic stenosis using quantitative magnetic resonance imaging

**DOI:** 10.1186/1532-429X-13-S1-P209

**Published:** 2011-02-02

**Authors:** Erin Madriago, Ronald Wells, Michael Jerosch-Herold, Brian S Diggs, Stephen M Langley, David J Sahn, Daniel J Woodward, Michael Silberbach

**Affiliations:** 1 Stanford University, Palo Alto, CA, USA; 2 Oregon Health & Science University, Portland, OR, USA; 3 Brigham and Women's Hospital, Boston, MA, USA

## Objective

To quantify myocardial blood flow (MBF) in children with aortic stenosis (AS) using adenosine-induced hyperemia

## Background

AS is a relentless disease that may have a prolonged asymptomatic phase followed by a rapidly downhill course. Valve replacement is often postponed in children because of technical limitations. However, it is likely that children with even mild AS have an abnormal cardiac microcirculation that contributes to lifelong morbidity and early death.

## Method

Children with mild or moderate AS and no coronary stenoses (mean Doppler pressure gradient 20.3 ± 10.4 torr ,n=11) were compared to a control group with hemodynamically normal hearts (“C”, pressure gradient 3.2 ± 1.5 torr, n=20). Mean age was 8.4 ± 4.4 years. For resting rest and hyperemia acquisitions the LV myocardium was divided into two slices (apical and basal) and each slice into six regions. The time course of average regional signal intensity during contrast transit was used to quantify absolute flow (ml/g/min) by deconvolution of the tissue curves with the arterial input of contrast. A linear mixed effects statistical model was employed to account for inter-subject variance (AS Vs C) and intra-subject variance (resting/adenosine, slice, and region), and also included interaction terms between AS and C, and treatment.

## Results

LV mass was significantly greater in AS than C (64.8 ± 13 Vs 48.9 ± 14 gm/m2, p =0.01). Age, body weight, LV volume and cardiac index were not different between the groups. Adenosine infusion increased heart rate and decreased blood pressure by ~ 15%, however there was no difference between AS and C. While adenosine increased MBF in all regions in both slices (p< 0.0001) the absolute increase in the AS group was significantly less than C (1.02 ± 0.10 Vs 1.52 ± 0.09 ml/gm/min, p <0.0001). The myocardial perfusion reserve (adenosine/resting flow) was significantly lower in AS compared to C (2.006 ± 0.5 Vs 2.625 ± 0.962, p= 0.024) due to both higher MBF at rest and a lower MBF during adenosine in the AS group. In the AS group gadolinium late enhancement was not observed.

## Conclusion

Adenosine-induced MBF is impaired in children with mild/moderate AS. These data suggest that improved surgical and catheter techniques to relieve afterload obstruction in younger children would be beneficial. Innovative medical strategies that induce cardiac angiogenesis in childhood may alter the long-term adverse effects of the abnormal microcirculation in AS.

